# Targets Involved in the Anti-Cancer Activity of Quercetin in Breast, Colorectal and Liver Neoplasms

**DOI:** 10.3390/ijms24032952

**Published:** 2023-02-02

**Authors:** Alessandro Maugeri, Antonella Calderaro, Giuseppe Tancredi Patanè, Michele Navarra, Davide Barreca, Santa Cirmi, Maria Rosa Felice

**Affiliations:** 1 Department of Chemical, Biological, Pharmaceutical and Environmental Sciences, University of Messina, 98166 Messina, Italy; 2 Department of Health Sciences, University “Magna Græcia” of Catanzaro, 88100 Catanzaro, Italy

**Keywords:** quercetin, cancer, hepatocellular cancer, liver cancer, colorectal cancer, breast cancer, flavonol, molecular target, polyphenols

## Abstract

Phytochemicals have long been effective partners in the fight against several diseases, including cancer. Among these, flavonoids are valuable allies for both cancer prevention and therapy since they are known to influence a large panel of tumor-related processes. Particularly, it was revealed that quercetin, one of the most common flavonoids, controls apoptosis and inhibits migration and proliferation, events essential for the development of cancer. In this review, we collected the evidence on the anti-cancer activity of quercetin exploring the network of interactions between this flavonol and the proteins responsible for cancer onset and progression focusing on breast, colorectal and liver cancers, owing to their high worldwide incidence. Moreover, quercetin proved to be also a potentiating agent able to push further the anti-cancer activity of common employed anti-neoplastic agents, thus allowing to lower their dosages and, above all, to sensitize again resistant cancer cells. Finally, novel approaches to delivery systems can enhance quercetin’s pharmacokinetics, thus boosting its great potentiality even further. Overall, quercetin has a lot of promise, given its multi-target potentiality; thus, more research is strongly encouraged to properly define its pharmaco-toxicological profile and evaluate its potential for usage in adjuvant and chemoprevention therapy.

## 1. Introduction

Phytochemicals are acknowledged for their beneficial effects in the fight against several diseases, including cancer [1,2,3]. Among these, flavonoids are the most representative ones, being the key elements of many dietary plans [4]. This is because they are endowed with a wide plethora of beneficial effects, which can be exploited in the management of various diseases and conditions, such as inflammation [5], infections [6], aging [7] and cancer [8]. In this context, flavonoids target several mechanisms affecting tumorigenesis [9,10], aiming at both genetic [11] and environmental causes [12,13,14]. Firstly, flavonoids are recognized worldwide for their antioxidant and radical scavenging activities, due to their chelating activity, exploitable in different conditions [15,16]. Moreover, flavonoids work as anti-cancer drugs inhibiting both chemokines and cytokines via immune cell modulation, as these elements are implicated in both cancer growth and metastasis [17]. By altering adhesion molecules, such as metalloproteinases, flavonoids have been shown to effectively reduce cancer metastatic factors [18,19,20]. One of the key traits that malignant cells acquire is the ability to escape from apoptosis, and flavonoids have the ability to thwart this process by inhibiting the action of caspases and B-cell lymphoma 2 (Bcl-2) family members [21,22]. Additionally, altered cell cycle progression is a key element in tumor formation, and flavonoids have been widely explored in this field, with their ability to impact the expression of various cyclin isoforms involved in each phase of the cell cycle [23,24]. Quercetin, flavonol abundant in a wide variety of plants [25], deserves to be considered the leading molecule of the whole flavonoid class, hence being part of the daily intake, by diet or supplementation, of each person worldwide [26]. This is due to its undoubted pharmacological properties ranging from anti-inflammatory [27], antioxidant [28], neuroprotective [29] to, nevertheless, anti-cancer ones [30].

The second leading cause of death worldwide is cancer. Indeed, 19.3 million new cases were documented in 2020, along with 10 million deaths caused by cancer [31]. In particular, the most representative neoplasms affect breast, colorectum and liver, accounting for the 11.7%, 10% and 4.7% of the worldwide incidence, respectively [31]. Despite the ever-growing strategies studied and established to treat these types of cancers, scientific community’s attention is always focused on the constant seeking of novel agent to employ in the management of cancer. Notably, natural compounds, endowed with multi-target capacity, thus simultaneously aiming at different molecular targets, are of great interest in pathologies arising from a cluster of events [32,33], and flavonoids have proved to excel in this field [34].

In this review, the role of quercetin in targeting the most important pathways involved in the type of neoplasms mentioned above is reported.

## 2. Quercetin and Breast Cancer (BC)

### 2.1. Quercetin Free Form in BC Experimental Models

For several decades, quercetin has been studied as an anti-tumoral molecule acting against breast cancer (BC), yet the concentrations and pathways involved in its beneficial effects are difficult to be established, owing to the heterogeneity of BC cell line genotypes used. From the initial studies, it was evident that quercetin could be useful in treatment of this form of cancer. In fact, using MDA-MB-468 cell line, quercetin at the concentration of 7 µg/mL (corresponding to 23 µM) arrested cells in G2/M phase [35] and showed growth inhibition with an IC_50_ of 55 µM [36]. Indeed, by using MCF-7 cell line, it was demonstrated that quercetin decreased cell viability with an IC_50_ of 17.2 µM [37] or 4.9 µM, contrasting pro-proliferative effect exerted by E2 and tumor necrosis factor alpha (TNF-α) [38]. The low concentration of quercetin necessary to inhibit growth of MCF-7 was also confirmed by Choi et al. (range concentration 1–20 µM) who observed apoptosis and arrest of cells in G2/M phase dependent on p21 protein [39].

With the increase in interest towards this flavonol, it was observed, by using the same cell line, that the concentration of quercetin needed to decrease cell viability was higher with respect to the results reported above (48 µM), along with an increase in reactive oxygen species (ROS) [40]. These results were confirmed by Lee et al. who also reported apoptosis induction at 100 µM. Investigating the mechanism, they also demonstrated that quercetin-mediated ROS generation triggered AMP-activated protein kinase (AMPK) activation and this reflected on cyclooxygenase 2 (COX-2) protein level that decreased, as quercetin concentration increased, in AMPK-dependent manner [41]. Van the Woude et al., using MCF-7 cell line for the first time, reported that quercetin effect was concentration-dependent, therefore high quercetin concentrations (maximum tested 100 µM) decreased cell viability, while lower ones (between 10 and 70 µM) had a strong pro-proliferative effect [42]. These initial observations were successively confirmed by another study in which estrogen receptor (ER)^+^ (MCF7, T47D) and ER^−^ (MDA-MB-231, HCC-38) cell lines were used and, considering cellular proliferation, it was demonstrated that quercetin had different effects, dependent on ER expression. In ER^+^ cells, a biphasic response to quercetin treatment was evident, with lower concentrations (1–60 µM) leading to pro-proliferative effect, while at higher ones, a decrease in cell viability was observed. Different was the response of ER^−^ cell lines, in which a decrease in proliferation was observed; however, it was from 0.1 µM [43]. Partially consistent with previous results, Xu et al. demonstrated that MCF-7 cells treatment with low concentration of quercetin (5–20 µM) led to pro-proliferative effect, upregulating ER membrane protein and, in contrast to the results reported above, also for MDA-MB-231 cells, a slight increase in cell number was observed. Instead, at higher concentration (100 µM), quercetin exerted anti-proliferative effect in both cell lines by cell cycle arrest and induction of apoptosis [44].

Moreover, anti-metastatic properties of quercetin were also reported. Particularly interesting was the antagonizing effect of quercetin, at the concentration of 5 µM, exerted on tamoxifen (5 µM), a standard chemotherapeutic drug, resulting in abrogation of its anti-cancer properties that were restored. This, in turn, resulted in a synergistic response by co-treatment with 100 µM quercetin. In this context, the observation that 100 µM quercetin treatment of MCF-7 cell line leads to a rapid induction of c-Fos (1 h) is perfectly in accordance with the view that c-Fos is a transcription factor associated to cellular proliferation. The authors demonstrated that its transcription depended on mitogen-activated protein kinase kinases (MEKs) and extracellular signal-regulated kinase 1/2 (ERK1/2) activation, while phosphoinositide 3-kinase (PI3K) was demonstrated not to be involved. Noteworthy, ER is not indispensable to induce estrogen or phytoestrogen-mediated c-Fos upregulation, as demonstrated by the use of the ER^−^ SK-Br-3 cell line. Treatment with quercetin or E2 was able to induce c-Fos by the involvement of another protein, G-protein-coupled estrogen receptor 1 (GPR30) that is expressed in this cell line. As the authors demonstrated, after the interaction of quercetin or E2 with GPR30, epithelial growth factor receptor (EGFR) was activated which, in turn, activated mitogen-activated protein kinases (MAPKs), triggering the cascade of events that also involved ETS transcription factor ELK1 (ELK1) and that led to c-Fos expression [45].

In contrast to the pro-proliferative effect reported above, treatment of MCF-7 cell line with low concentrations of quercetin (0.1 nM to 1000 nM) has highlighted an increase in phosphatase and tensin homolog (PTEN) protein level with concomitant decrease in phosphorylated protein kinase B (AKT) that reflected on an increase in p27 and arrest of cell cycle [46]. Regarding support of negative regulation by quercetin on PI3K/AKT pathway, another study using HCC1937 (PTEN^−/−^) and T47D (PTEN^+/+^) cell lines has demonstrated that quercetin 25 µM led rapidly to reduction in phosphorylated AKT level, even when its activation was stimulated by epithelial growth factor (EGF), as in T47D cell line, indicating that the reduction of AKT activation was not strictly linked to PTEN, but probably to an inhibitory effect exerted by other mechanisms [47]. In agreement with a negative effect of quercetin on PI3K/AKT/mammalian target of rapamycin (mTOR) pathway activity, the study of Jia et al., which used MCF-7 and MDA-MB-231 cell lines, observed that treatment with 30 µM of quercetin decreased cellular invasion and migration capacities by decreasing AKT/mTOR activity, leading to autophagy induction. This reflected on reduction in glucose cellular concentration linked to decreased glucose transporter 1 (GLUT1) and reduction in pyruvate kinase M2 (PKM2) and lactate dehydrogenase A (LDHA) protein levels. Administration of 50 mg/kg of quercetin to tumor xenograft animal model led to very similar results with decreased tumor volume and reduced levels of markers related to tumor growth and metastatic abilities [48]. The results obtained by Jia et al. can also be interpreted in various ways because it was reported that PKM2 is overexpressed in BC tissues, and this correlates with increased activation of AKT caused by PKM2-mediated phosphorylation of mTOR complex 1 (mTORC1) inhibitor, AKT1S1, and reduced autophagy [49]. The importance of quercetin-mediated effect on PI3K/AKT/mTOR pathway was also demonstrated in a study in which BC stem cells (BCSC) resistant to chemotherapeutic molecules and radiotherapy were used. Indeed, treatment of CD44^+^ BCSC isolated from MCF-7 cells with quercetin 50 µM caused reduction in cell viability and metastatic properties. Results were also confirmed in vivo by xenograft mouse model, and the mechanism that was assumed to occur, based on experimental data, is that quercetin treatment decreased protein levels and phosphorylated forms of PI3K, AKT, and mTOR [50]. The different results reported in the literature can be explained considering the heterogeneity of cell lines that sometimes respond differently to the treatment even if they share the same characteristic, as demonstrated by a study conducted on a panel of nine triple negative BC (TNBC) cell lines (HCC1806, HCC70, HCC1937, BT-549, BT-20, Hs578T, MDA-MB231, MDA-MB 157 and MDA-MB-468 BT-549). Treatment with different quercetin concentrations demonstrated that the 200 µM led to migration inhibition and decreased invasion abilities. Considering that these processes involve different kinases that are activated/inactivated by phosphorylation, the authors used a Phospho-Kinase array, confirming the results in cells in ab attempt to generate a general cellular response to quercetin that would be common to the different cell lines. Following this criterion, it was demonstrated that quercetin treatment decreases AKT phosphorylation, leading to reduced activity of downstream target proteins, including glycogen synthase kinase 3 alpha/beta (GSK3α/β) and WNK lysine deficient protein kinase 1 (WNK-1), a protein that in other cancer types is linked to epithelial–mesenchymal transition (EMT) promotion. Moreover, β-catenin, ERK1/2, c-JUN N-terminal kinase (JNK) 1/2/3, and p38α resulted less phosphorylated, indicating that quercetin can also reduce their activity while increasing checkpoint kinase 2 (CHK2) phosphorylation, whose activity is inversely correlated with cellular migration, indicating that quercetin can probably influence cellular response acting on different target proteins [51].

A kinase that seems not to be regulated in any of TNBC cell lines considered in the study of Shahi Thakuri et al. is AMPK, in contrast with several studies in which an increase in its activity was observed. For example, it was reported, using MDA-MB-231 and MDA-MB-435 cell lines, that quercetin treatment at the concentration of 15 µM increased phosphorylation level of AMPK and decreased AKT activity strongly in both cell lines, independently of cell genotype. This kinase response to this flavonol is believed to be at the base of growth inhibition observed after 48 h of treatment, linked to arrest in G2/M phase of cell cycle, and not to an apoptosis increase, since in the same interval of time considered, this was not statistically significant [52]. Despite the very interesting results, the authors did not investigate more in depth the mechanism by which quercetin activated AMPK that can be dependent on different cellular conditions such as oxidative stress, DNA damage, compromised energy cellular status, as well as triggered cells towards apoptosis or autophagy [53]. Quercetin-mediated AMPK activation could also be at the base of the observation that in MDA-MB-231 and MDA-MB-157 cell lines, treatment with quercetin brought a reduction in lipid synthesis and increased in cancer cells by inhibition of fatty acid synthase (FAS) enzyme, and this reflected on the decrease in cell viability and increase in apoptosis, as well as β-catenin decrease [54,55,56].

Although it is considered that activated AMPK can inactivate AKT, this conclusion is still controversial. In this regard, Lee and Park demonstrated that quercetin treatment in MCF-7 cell line led to increased level of phosphorylated AMPK and decreased level of phosphorylated AKT, in a concentration-dependent manner but by use of specific inhibitors of AKT or AMPK. It was demonstrated that the two kinases influence each other despite being independent, a condition more evident for AKT, whose phosphorylation decreased after quercetin treatment even if AMPK activity was inhibited, suggesting a more complex mechanism of regulation [57].

Apart from AKT pathway, JNK can be also involved in quercetin-mediated anti-proliferative effect observed in BC cells by regulation of forkhead box O3A (FOXO3a) transcription factor activity, as demonstrated by using MDA-MB-231 cell line. Treatment with quercetin at the concentration of 20 µM decreased cell viability and increased apoptosis rate and cell cycle growth arrest and cellular responses, as demonstrated by the authors, which is linked to JNK-mediated FOXO3a increased protein level and nuclear translocation [58].

In the overall literature related to quercetin anti-cancer activity, microRNA (miRNA) expression was little considered, even if today it is known that non-coding RNAs (ncRNAs) are very important for cellular homeostasis and that their dysregulation is always related to cancer. Indeed, by using MCF-7 and MDA-MB-231 cell lines, as well as tumor xenograft mouse model, it was demonstrated that quercetin treatment increased miR-146a expression in a concentration-dependent manner and that it was involved in apoptosis induction by negative regulation of EGFR expression, overexpressed in BC due to gene amplification [59].

Besides signaling pathways that can be modulated by quercetin, this flavonol can influence the expression of different proteins as demonstrated by using MDA-MB-231 and MCF-7 cell lines, in which treatment with quercetin 100 µM reduced protein levels of both heat shock protein (HSP) 70 and HSP27 not dependent on minor binding of heat shock factor (HSF) transcription factor to their promoters [60]. These results were also confirmed by Kryga et al., who observed, by using the same cell lines, a decrease in HSP90 protein level, a chaperone very important in assisting oncogenic protein in post-translational maturation, such as AKT, and it was reported that its inhibition is related to strong breast anti-tumor effects [61,62]. Accordingly, quercetin-mediated downregulation of HSP90 correlated with apoptosis induction, even if higher quercetin concentrations were needed for MDA-MB-231 with respect to MCF-7 cell line to produce the same effect [61]. The quercetin-mediated downregulation of HSP27 was also demonstrated to be important in BCSC self-renewal and metastatic properties, as described by Wei et al., (2011) when using BCSC ALDH^+^ AS-B145 and AS-B244 cell lines. Indeed, quercetin treatment (0–200 µM) reduced HSP27 protein level in both cell lines in a concentration-dependent manner, and also reduced the population of ALDH^+^ cells, the dimension of mammospheres, cell migration capacity and EMT in an HSP27-dependent manner. Moreover, HSP27 inhibition led to minor nuclear translocation of NF-κB linked to decrease in proteasomal degradation of HSP27-mediated NF-κB inhibitor (IκBα), even if the role of quercetin in this process was not analyzed [63].

Another protein whose expression can be regulated by quercetin is urokinase, which is highly expressed in BC. It was demonstrated that quercetin treatment in MDA-MB-231 cell line at the concentration of 45 µg/mL (150 µM), which exerted the maximum growth inhibition, led to a decrease in intracellular calcium concentration, probably caused by the increase in its cellular export that negatively regulated urokinase activity, probably dependent on PKC decreased activity mediated by quercetin, as hypothesized by the authors [64].

Phosphofructokinase platelet-type (PFKP) protein, highly expressed in BC and associated with aerobic glycolysis, is another protein whose expression can be regulated by quercetin. Using meta-analysis software of public dataset, a correlation between PFKP high expression was highlighted, associated principally with TNBC type and poor patients’ prognosis. To better understand the role of PFKP in aerobic glycolysis and the potential beneficial effect of quercetin, TNBC MDA-MB-231 cell line, treated with the flavonol at the concentration of 25 µM, showed reduced PFKP and lactate dehydrogenase A (LDHA) protein levels that reflected on minor cellular invasiveness and migration abilities [65]. The observed decrease in PFKP protein level could be in part justified assuming that quercetin inhibits AKT activity. In fact, it was reported that AKT-dependent phosphorylation of PFKP at S386 is important as it competes with the binding of tripartite motif containing 21 (TRIM21) E3 ligase, reducing its proteasomal degradation [66].

The evidence gathered on the anti-cancer effects of quercetin in BC experimental models is reported in Table 1.

### 2.2. Effects of Combination of First-Line Treatments with Quercetin in BC Experimental Models

In addition to the beneficial effect of quercetin used alone, it can enhance the efficacy of other molecules in BC as was demonstrated for docetaxel, whose cytotoxic activity was synergistically potentiated by quercetin (40 µM) in MCF7-DR (drug resistance) where it led to the decrease in lymphoid enhancer-binding factor 1 (Lef1), a protein strongly associated with cell drug resistance. As a consequence, docetaxel intracellular concentration was increased [67]. Moreover, it was demonstrated that docetaxel and quercetin can act synergistically (7 nM and 95 µM, respectively) on MDA-MB-231 cell line in apoptosis induction by a mechanism in which p53 and BCL2-associated X protein (BAX) expression was increased, while pAKT, pERK1/2, phospho-signal transducer and activator of transcription 3 (pSTAT3) protein level were decreased [68].

Regarding support of beneficial effects of combined use, quercetin co-administration with cisplatin to EMT6 tumor xenograft mouse model demonstrated to be more efficient compared to cisplatin alone in reduction in cancer size and cisplatin-mediated breast cytotoxic effects [69].

Another example of synergism was observed when MDA-MB-231 cell line was treated with 5-fluorouracil and quercetin, which not only decreased cell viability but also inhibited cell migration, as demonstrated by the decreased expression of matrix metallopeptidase (MMP)-2 and -9 [70]. Results partly confirmed that when MCF-7 cell line was used, 5-fluorouracil and quercetin synergistically increased apoptotic rate [71].

The synergistic effects of quercetin observed with anti-neoplastic drugs were also demonstrated for human recombinant tumor necrosis factor-related apoptosis-inducing ligand (rhTRAIL) by using MCF-7 and BT-20 cell lines (TRAIL resistant), as it was observed that quercetin, at the maximum concentration of 50 µM, enhanced rhTRAIL pro-apoptotic effects by inducing proteasomal degradation after 72 h of treatment of CASP8 and FADD-like apoptosis regulator (c-FLIP), a negative regulator of extrinsic apoptosis, and increasing expression of Death Receptor 5 (DR5) by a mechanism that was not investigated by the authors [72].

Another example of the beneficial effect of quercetin against BC chemoresistance is represented by flavonol-mediated enhancement of cytotoxic effect of paclitaxel, whose cell resistance is linked essentially to a major export rate caused by overexpression of P-glycoprotein that was shown to be downregulated by quercetin at the maximum concentration of 10 µM, as demonstrated in MCF-7 cell line resistant to doxorubicin (MCF-7/ADR) [73]. These results were also confirmed in another study on the same cell lines, but also in BCSC, where it was demonstrated that quercetin (0.7 µM) could enhance the cytotoxic effect of doxorubicin, paclitaxel and vincristine by decreasing P-glycoprotein protein level and reducing nuclear translocation of Y-box binding protein 1 (YB-1) [74]. This is a transcription factor highly expressed in BC involved in transcription of genes linked to cell proliferation and drug resistance that is translocated in the nucleus after AKT/mTOR or p90RSK-mediated phosphorylation, suggesting that the results obtained by Li et al., (2018) could be linked to quercetin-mediated inhibition of AKT/mTOR pathway [48].

The evidence collected on the synergistic effects of quercetin in BC experimental models is reported in Table 2.

### 2.3. Delivery Systems for Quercetin in BC Experimental Models

Quercetin, albeit being effective as anti-tumor molecule, has limitations of applicability linked to its stability for in vivo use; in fact, it loses part of its properties in the gastrointestinal tract, and this has encouraged researchers to develop new strategies of delivery systems.

The mechanism of quercetin-mediated P-glycoprotein downregulation was also observed when paclitaxel was encapsulated with quercetin in mesoporous silica nanoparticles coated with chondroitin sulfate (MSNs) (MSNs-ChS@PQ) and used in MCF-7/ADR cell line. The treatment enhanced paclitaxel cytotoxic effect, probably owing to drug increased cellular concentration, inducing growth inhibition and apoptosis. These results were confirmed also in vivo by tumor xenograft animal model, in which no damage to other organs was observed [75].

The beneficial effect of therapy based on combination of quercetin with chemotherapeutic molecules, such as doxorubicin, was also demonstrated by using mixed micelles formed by hyaluronic acid-based conjugate and d-α-tocopheryl poly-(ethylene glycol) 1000 succinate loaded separately with quercetin or doxorubicin that were tested on the drug-resistant cell line MDA-MB-231/MDR1 in successive combinations. This strategy of pre-treatment of mixed micelles loaded with quercetin followed by treatment with those loaded with doxorubicin more efficiently induced apoptosis compared to single treatments or the respective free forms of the molecules, and it was demonstrated on tumor xenograft mouse model that the system was also efficient in vivo [76].

A delivery system based on nanoparticles formed by quercetin and regenerated silk fibroin (RSF) and coated with the nonapeptide LyP-1 (LyP-1-QU-NPs) was used on 4T1 cell line after characterization of structure, stability, and drug release kinetic in different experimental conditions, confirming its adaptability to tumoral microenvironment. To better define the effectiveness and specificity of LyP-1 for BC cells, 4T1 cell line was used since its high expression level of p32 that interacts with LyP-1. In this model, it was demonstrated that, compared to quercetin free form, LyP-1-QU-NPs more efficiently decreased cell viability by apoptosis induction and arrest in G2/M phase, as well as reduced glycolysis rate, migration and invasion abilities, probably because of a better uptake and mitochondrial accumulation that damaged them. The in vitro results were confirmed in vivo by 4T1 tumor xenograft mouse model, where authors reported great accumulation of LyP-1-QU-NPs at the tumor site that reflected on reduced tumor volume, anti-proliferative ability, and apoptosis induction. Moreover, in this type of BC, nanoparticles led also to lung metastasis reduction and signs of damage were not observed in other organs [77]. The results reported in this study are very interesting and the fact that p32 protein is expressed also in other BC cell lines, such as MDA-MB-465, MDA-MB-231, BT549, MCF7, make this delivery system very promising [78].

Another strategy was to use nanoparticles of apoferritin whose cavity was loaded with quercetin and curcumin. When tested in MCF-7 cell line, this system more efficiently showed an increase in cellular ROS production and apoptosis induction compared to the two molecules used singularly [79], the same effects reported by the use of another delivery system tested on the same cell line that consisted in quercetin encapsulation in solid lipid nanoparticles, a system that increases stability of the complex in blood circulation and has high drug-loading capacity [80].

Another system designed to target BC cells was based on nanoparticles formed by polylactic-co-glycolic acid (PLGA), linked to polyethyleneimine (PEI) to which hyaluronic acid (HA) was bound, containing quercetin and docetaxel (PP-HA/NPs) [81]. This delivery system was used in 4T1 cell line and provided better results with respect to treatment with quercetin and docetaxel, used singularly or in combination as a free-form, as well as in different partial nanoparticle formulation. In particular, PP-HA/NPs was more efficient in growth inhibition, apoptosis induction, and reduction in invasion and migration capacities, probably linked to increased cellular uptake due to interaction between CD44^+^ membrane protein and HA present on nanoparticles surface. At molecular level, a decrease in phosphorylated AKT and MMP-9 protein level was observed, which is probably linked to the effect of AKT decreased activity on NF-κB, as demonstrated in other cancer types [82,83]. Of particular interest is that in vivo study demonstrated a great accumulation of PP-HA/NPs in primary tumor and pulmonary metastasis [81].

Another delivery system, consisting in quercetin-conjugated gold nanoparticles (AuNPs-Qu-5), was used in MCF-7 and MDA-MB-231 cell lines that showed decreased cell viability compared to free quercetin in both cell lines. Notably, AuNPs-Qu-5 treatment decreased EMT, migration and invasion abilities of cancer cells, as well as angiogenesis using in HUVEC cell line. Analysis of PI3K/AKT pathway demonstrated that it was strongly inhibited by AuNPs-Qu-5 delivery system, probably triggered by reduction in EGFR activity [84]. Moreover, AuNPs-Qu-5 system was also used in 7,12-dimethyl benz(a)anthracene (DMBA)-induced BC rat in which a decrease in tumor volume as well as very similar architecture, compared to normal tissue, of epithelial mammary gland was reported [85].

**Figure 1:**
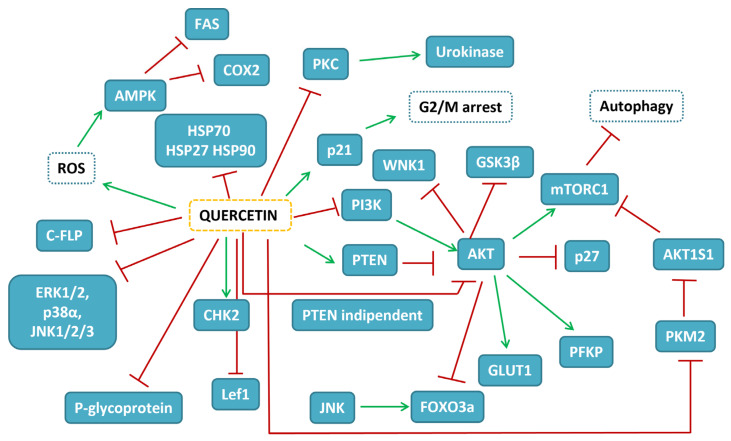
Map of the interactions of quercetin in BC experimental models. In blue-filled boxes, the protein influenced by quercetin treatment are reported, while blue-dashed boxes indicate cellular events. Red T-shaped lines correspond to inhibition, while green arrows to activation.

Research on the potentiality of different delivery systems of quercetin in BC experimental models is reported in Table 3. The map of the pathways influenced by quercetin in BC experimental models is depicted in Figure 1.

## 3. Quercetin and Colorectal Cancer (CRC)

### 3.1. Quercetin Free Form in CRC Experimental Models

Quercetin anti-tumoral effects against colorectal cancer (CRC) have been an object of study for approximately 30 years and, to this day, the exact mechanism of action is not completely understood. Several cell lines and few animal models were used and, in the literature, contradicting results are reported, depending on experimental conditions. For example, it was demonstrated that quercetin can be unstable in the culture media used, because it tends to autoxidize, thus leading to H_2_O_2_ production [86]; it also tends to precipitate at high concentrations [42], and this is dependent on medium composition. Another problem is linked to cell lines used, as in the case of SW480 cells that showed higher sensitivity to growth inhibition of quercetin (IC_50_ about 60 µM) respect to mouse CRC clone 26 cell line (IC_50_ > 160 µM). This different effect was also highlighted by the analysis of cell cycle phases that showed an increase in SW480 cells in G2/M phase, while for clone 26, even if a slight increase was also observed, it was less evident. In the same manner, apoptosis rate was also influenced and followed the same trend in the range of concentrations considered. A deeper analysis has shown that quercetin treatment of SW480 cells reduced the transcription of β-catenin-mediated genes in a concentration-dependent manner [87]. Using Caco-2 cell line, it was highlighted that quercetin treatment reduced cell proliferation with an IC_50_ of approximately 50 µM and, considering 5 and 50 µM concentrations, downregulated mRNAs related to cell cycle progression, as well as an increase in the number of cells in sub-G1 phase [88]. Contrasting results were reported for HT-29 cell line; in fact, Agullo et al. demonstrated that cell growth is inhibited by quercetin with IC_50_ of 15 µM [86], while van der Woude et al., using the same cell line, observed that at lower concentration, cells proliferate rather than are inhibited by quercetin (from 0 to 70 µM) [42]. Similarly, the pro-proliferative effect was also observed in HCT-116 cell line, but from 0 to 20 µM [42]. On the other hand, using HT-29, COLO205, COLO320-HSR, and COLO205-X (derived from mouse induced tumor with COLO205) cell lines, quercetin (200 µM) was effective in reducing cell viability of HT-29 (about 50%) and COLO 205 (about 25%) but had no effect on COLO320-HSR cell lines [89].

Partially, in accordance with the results reported above, quercetin treatment of HT-29 cell line led to growth inhibition of approximately 50% and chromatin condensation at 100 µM, concentration at which the authors observed an increase in apoptosis and arrest in the G1 phase of cell cycle after 24 h. The anti-proliferative effect exerted by quercetin was associated to an AMPK activation, a kinase that responds to different stimuli and can trigger cells versus apoptosis by Tuberous Sclerosis Complex 1/2 (TSC1/2)-mediated mTORC1 and AKT inhibition [53,90]. In fact, consistent with AMPK activation, increase in pro-apoptotic and decrease in anti-apoptotic protein levels were reported. The in vitro results were also partly confirmed in vivo by HT-29 tumor xenograft mouse model in which quercetin administration (50 or 100 mg/kg) reduced tumor volume probably by the same mechanism of apoptosis induction [91]. AMPK quercetin-dependent activation was also demonstrated by Lee et al., who, using HT-29 cell line, have observed that its activation led to COX-2 protein level decrease and apoptosis [41], and COX-2 inhibition was also demonstrated in the same cell line by other authors that observed also an increase in IκBα expression [92]. In support of quercetin-mediated inhibition of NF-κB activity, Zhang et al. demonstrated that flavonol treatment, which inhibited the growth of Caco-2 and SW620 cell lines with IC_50_ values of 35 μM and 20 μM, respectively, induced caspase-dependent apoptosis in a concentration-dependent manner, probably by negative regulation of NF-κB activity, as demonstrated by reduced p65 and IκBα phosphorylated protein levels and increased IκBα expression [93]. These results can be explained considering that the expression of COX-2, which is highly upregulated in CRC, is mediated by NF-κB transcription factor that is positively regulated by AKT, whose activity is negatively controlled by AMPK [94,95]. The AMPK activation can also be linked to ROS-mediated sestrin2 overexpression, a protein that is expressed at low level in CRC. Kim et al. demonstrated that quercetin treatment of HCT116 (p53 wild type) and HT-29 (p53 mutated) cell lines at both 25 and 50 µM caused ROS production that induced apoptosis by enhancing expression of sestrin2, independently from p53 protein [53,96,97]. An additional study using the same experimental condition reported above has more markedly highlighted the importance of sestrin2 by its silencing, and added an involvement, over AMPK activation, of p38MAPK in inducing apoptosis [98]. The results reported by Kim et al. seem to delineate two possible pathways involved in apoptosis induction mediated by sestrin2 activation (AKT/mTOR and p38), and the data reported suggest that both, singularly and in combination, are essential to induce apoptosis. A possible explanation is that p38MAPK activation can increase transcription of PTEN, a negative regulator of PI3K/AKT/mTOR pathway, by activation and nuclear translocation of FOXO3a that was demonstrated to be involved in PTEN transcription [99,100]. P38MAPK can also be activated by quercetin treatment through estrogen receptor β1 (ERβ1) as was demonstrated by using DLD-1 cell lines that express only this estrogen receptor. The authors demonstrated that quercetin treatment induced apoptosis by activation of ERβ1 that led, with the same efficiency of E2, to increased p38MAPK phosphorylated protein level, which is positively involved in tumor suppressor PTEN expression. The increased level of PIP3,4,5 phosphatase, PTEN, can justify the lack of PI3K/AKT/mTOR pathway activation, while no evident involvement of ERK1/ERK2 was observed [101].

Although the results reported above highlight a positive role of quercetin in activation of AMPK, in the same period, Kim et al. reported contrasting data, both by in vivo and in vitro experiments. Using HCT116 tumor xenograft mouse model, they observed that administration of 50 mg/kg of quercetin reduced tumor volume and the protein level of AMPK phosphorylated form. To better understand the mechanism and recreate conditions that resemble tumor microenvironment, they confirmed the in vivo results using HCT116 cell line grown both in normoxia and hypoxia (0.1% O_2_) conditions. In fact, cell treatment with quercetin at the concentration of 100 µM induced apoptosis dependent on p53 functionality, and AMPK activity inhibition was more evident in hypoxic condition compared to normoxia, in which level variations of phosphorylated form of AMPK were less evident. As the authors demonstrated, this was probably dependent on two factors that have opposite effects: cellular AMP increase and direct inhibition of its activity. AMPK inactivation also resulted crucial in the regulation of hypoxia inducible factor 1 subunit alpha (HIF-1α) activity that was strongly upregulated in hypoxia while its expression decreased in function of quercetin concentration increase. Moreover, in hypoxic condition, AMPK inhibition also led to increased cellular sensitivity to cisplatin and etoposide, suggesting AMPK as potential therapeutic target in inducing tumor cell death [102].

Apart from the results reported by Kim et al., (2012), a positive role of PI3K/AKT pathway in tumor progression was also demonstrated by Yang et al., who observed its involvement in expression of CSN6, a subunit of the constitutive photomorphogenesis 9 (COP9) signalosome (CSN) that regulates E3 ubiquitin ligases. Using HT-29 cell line, quercetin treatment led to growth inhibition (IC_50_ 81.65 ± 0.49 µM after 48 h) and induced strong cellular morphological changes and apoptosis in a concentration-dependent manner. To demonstrate a role of CSN6 in pro-survival mechanisms, it was overexpressed in HT-29 cells, and to demonstrate that, CSN6 upregulated c-Myc and Bcl-2 protein levels, while p53, Bax and cleaved caspase-3 were downregulated, indicating that CSN6, positively regulated by AKT, controlled the expression of these proteins [103]. What was not commented on by the authors is that even if CSN6 is overexpressed, its protein level results sensible to quercetin treatment, suggesting a post-translational mechanism triggered by flavonol that reflects on all CSN6 target proteins. An additional confirmation of the negative role exerted by PI3K/AKT pathway in apoptosis induction was obtained by the observation that quercetin treatment led to decrease in ErbB2 (HER2) and 3 (HER3) protein levels with consequent decrease in PI3K activation. For this, HT-29 and SW480 cell lines were used which were growth-inhibited by quercetin with IC_50_ of approximately 100 µM [104].

The complexity of CRC cell lines response to quercetin is augmented if the study of Refolo et al., in which it was demonstrated that a relatively low concentration of this flavonol (50 µM) upregulated specifically the cannabinoid receptor 1 (CB1-R) expression in Caco-2 and DLD-1 cell lines at a concentration in which quercetin exerted growth inhibition by apoptosis induction. Analyzing different pathways, it was demonstrated that quercetin, probably in part interacting with CB1-R, inhibited proliferation by decrease in PI3K/AKT activity, while JNK and c-Jun were both activated. Moreover, β-catenin nuclear translocation was inhibited by its increased degradation, as demonstrated previously by Park et al., using the same quercetin concentration on SW480 CRC cell line, even if the mechanism was not investigated by the authors [105]. On the contrary, the study of Refolo et al. highlighted that its degradation is partly CB1-R-mediated and furthermore demonstrated that the activation of the same receptor is also involved in cell migration inhibition [106]. The interaction of quercetin with CB1-R with consequent anti-proliferative effects was also investigated in vivo using CRC-induced mouse model, in which mice were fed with 0.5% quercetin during CRC induction and, also in this case, increase in CB1-R protein level was observed in normal mice and even more strongly it was observed in quercetin-treated CRC-induced mice [106]. Moreover, decrease in STAT3 protein level and of its phosphorylated form was highlighted, as well as increase in pro-apoptotic proteins in quercetin-treated CRC-induced mice that was not observed in normal mice, indicating that beneficial effects of quercetin are also related to anti-inflammatory activity [107]. This was confirmed by Lin et al. using a more severe phenotype of CRC-induced mouse model treated with quercetin (30 mg/Kg) during the cancer induction. The authors demonstrated that the administration of quercetin during CRC induction led to decrease in tumor number and size compared to untreated animals and, furthermore, it reactivated the altered hemopoietic system, probably by a general decrease in oxidative stress, suggesting the use of quercetin to prevent and contrast cancer development [108].

Moreover, the expression of different proteins can be influenced by quercetin, such as non-steroidal anti-inflammatory drug activated gene-1 (NAG-1) as demonstrated in HCT-116 and other different cancer cell lines. In these models, the expression of NAG-1 increased in a quercetin concentration-dependent manner (maximum tested 40 µM), and its transcription depended on Specificity protein 1 (SP1) and early growth response 1 (EGR-1) transcription factors that directly interacted with NAG-1 promoter even if with different efficiency, while quercetin-mediated activation of p53 that had a positive effect on NAG-1 transcription was not involved directly in binding to its promoter [109].

As reported above, COX-2 protein level, that is highly expressed in CRC cells and seems to be related to carcinogenesis is also influenced by quercetin. Using DLD-1 cell line, it was demonstrated that quercetin can reduce to basal level the TNF-alpha-stimulated COX-2 expression. The mechanism was not investigated but it is possible that it can involve PI3K/AKT or other pathways [110].

Even if research has focused for the most part on quercetin-induced growth inhibition and mechanisms of apoptosis induction, the study of Han et al. has demonstrated, using Caco-2 cell line, that quercetin at low concentrations (5 µM) was able to reduce cellular migration and invasion abilities by inhibition of Toll-like receptor-4 (TLR-4) and/or NF-κB activity that reflected on decreased inflammation, a hallmark of tumor cells [111].

Moreover, quercetin treatment can inhibit TGF-β1-induced EMT, as demonstrated by using SW480 cell line; in fact, quercetin 100 µM could reverse the pathological cellular transition as demonstrated by increase in E-cadherin, as well as decrease in vimentin and Twist1 transcription factor protein levels [112]. The downregulation of Twist1 mediated by quercetin was observed, probably linked to the inhibition of PI3K/AKT pathway that positively regulates NF-κB activity which is directly involved in induction of Twist1 expression [113].

Another factor that is rarely considered in experimental design is that different cell lines have mutations in several genes responsible of the uncontrolled growth of tumor cells; thus, the reported results are sometimes contradictory. For example, it was demonstrated that HCT-15 (KRAS G13D) and CO-115 (BRAF V600E) cell lines that have different gene mutations involving also phosphatidylinositol-4,5-bisphosphate 3-kinase catalytic subunit alpha (PIK3CA), PTEN and p53 are sensitive at almost the same manner to relative low quercetin concentrations (maximum tested 20 µM), showing reduction in cell viability and proliferation rate and apoptosis induction, probably triggered by inhibition of RAS and PI3K activity [114]. Moreover, using cell lines characterized by KRAS G13D (SW480, HCT116 and laboratory-created DLD-1^KRASG13D^) gene mutation, it was observed that when treated with quercetin at the maximum concentration of 100 µM, they were more sensitive to quercetin compared to KRAS wild type gene cell lines (DLD-1, COLO205, HT29, WIDR) and this major sensibility reflected on induction of extrinsic and intrinsic apoptosis, probably linked to inhibition of AKT and activation of JNK that has such an important role in this process view that its inactivation reverses the positive quercetin effects [115]. These results were in accordance with its role in apoptosis induction by different mechanisms as phosphorylation of BCL2-associated agonist of cell death (Bad) and Bcl-2 interacting mediator of cell death (Bim) or release of cytochrome c through a BH3 interacting domain death agonist (Bid)-Bax-dependent mechanism [116]. In support of this finding, very similar results were obtained on KRAS and HRAS G12V mutation, as it was demonstrated by Psahoulia et al., (2006) who correlated the presence of mutation in the different cell lines with major abilities of quercetin to reduce cell viability, in some cases by autophagy [117].

The strategy used have delineated the mechanism of quercetin action only in part, making evident the fact that new experimental approaches must be considered. Transcriptome analysis by RNA-Seq is an emerging technology that has the advantage of providing a lot of information relative to all the transcripts (coding and noncoding RNA) expressed by a tissue or cellular type and can also provide information about the genes that are differently expressed. In the landscape of the literature regarding quercetin, this new approach was rarely used, even if it can be more informative. A study of Pang et al. has compared deposited RNA-Seq data of colon cancer and rectal cancer patients to healthy persons, and considering the differentially expressed genes, they detected 912 upregulated and 754 downregulated genes for colon cancer, while for rectal cancer they reported 1120 upregulated and 1115 downregulated genes. Intersecting the in silico prediction of interacting protein with quercetin and differentially expressed genes with high log_2_ fold-change values, the authors selected a set of genes that quercetin can downregulate or upregulate directly. Moreover, analyzing genes that were strongly differently expressed and the survival rate of patients from which tissue was removed, it was possible to correlate up- and downregulated genes with diagnosis and prognosis prediction [118]. Another transcriptome study was conducted on HCT116 cell lines comparing control cells with quercetin-treated cells, and a big number of differently expressed genes was reported, part of them correlated to PI3K/AKT, MAPKs and Ras pathways according to KEGG analysis. Of particular importance is that the differential expression is not only linked to mRNA, but also involves miRNA (19 downregulated and 64 upregulated), ncRNA (89 downregulated and 151 upregulated) and circRNA (37 downregulated and 94 upregulated). Moreover, simulation of interconnection between them, like, for example, circRNA-miRNA-mRNA, has highlighted a delicate network of interaction of strong importance in the cancer physiology and provided the bases of data on the ways in which quercetin can exert its anti-cancer activity [119].

The evidence gathered on the anti-cancer effects of quercetin in CRC experimental models is reported in Table 4.

### 3.2. Effects of Combination of First-Line Treatments with Quercetin in CRC Experimental Models

Apart from the effects that quercetin can singularly exert as anti-cancer agent, it can also potentiate the properties of chemotherapeutic molecules. This was demonstrated by using HCT-15 cell line (p53 mutated), which is resistant to 5-fluorouracil treatment and high concentrations are needed in vitro to inhibit cell growth and induce apoptosis. Indeed, it was observed that co-treatment with 12 µM quercetin and 100 µM 5-fluorouracil enhanced, in additive manner, apoptosis rate compared to single treatment, even if the concentration necessary to have this effect was higher than for CO-115 cell lines (p53 wild-type) (1 µM 5-fluorouracil), for which the co-treatment with 12 µM quercetin led to a synergistic effect. Moreover, apoptosis induction was not influenced by caspases inhibitors, suggesting caspase-independent cell death but, in any case, dependent on functional p53 protein that could be at the base of the different cellular behavior and response to chemotherapeutic molecules [120]. The beneficial effects on apoptosis induction of quercetin, even if used at very high concentration (580 µM), and 5-fluorouracil combination was also demonstrated in another cell line, HT-29, characterized by a homozygous p53 (R273H) mutation that brings to canonical tumor suppressor loss of function and even acquires oncogene characteristic [121]. Although the authors determined an increase in p53 expression to which they related the positive effects observed, it is plausible that the reported decrease in AKT and mTOR and increase in PTEN and p38MAPK protein level is at the base of the decreased viability observed [122].

Quercetin (10 µM) was also used in combination with radiation using DLD-1 cell line and it was noted that the co-treatment reduced the surviving cell fraction. The results in DLD-1 tumor xenograft mouse model confirmed that treatment with quercetin (30 mg/kg) or radiation separately or in combination highlights that the combined therapy is more efficient in reducing volume and doubling time of tumor. Mechanistic studies using different cell lines revealed that the DNA double strand breaks persisted in co-treated cells compared to cells subjected to single treatment by inhibition of ATM activity, which probably explains the major sensitivity of cell and animal model to a combined therapy [123]. Quercetin can also enhance the cytotoxic effect of cisplatin in HT-29 cell line; the co-treatment with 50 µM flavonol and 10 mg/L cisplatin drastically reduced cell viability by induction of apoptosis and cell cycle arrest in G2/M phase by a mechanism that involved a quercetin-mediated downregulation of NF-κB [124]. Quercetin is also functional on CRC stem cells (CSC), a population of cells that is associated with tumorigenicity, metastasis and drug resistance. Using isolated CD133^+^ cells from a culture of HT-29, it was demonstrated that quercetin treatment enhanced the cytotoxic effect of doxorubicin used at lower concentration compared to when it was used separately by cell arrest in G2/M phase and induction of apoptosis that was more evident in HT-29 compared to CD133^+^ cells, allowing the use of lower concentrations of doxorubicin in an attempt to decrease the cytotoxic side effects on normal cells [125].

The synergistic effects of quercetin in CRC experimental models are presented in Table 5.

### 3.3. Delivery Systems for Quercetin in CRC Experimental Models

The encapsulation of quercetin and alantolactone in micelles of 1,2-distearoyl-sn-glycero-3-phosphoethanolamine-N-methoxy-polyethylene glycol 2000 (DSPEPEG2000) and D-α-tocopherol polyethylene glycol succinate (TPGS) (QA-M) used on microsatellite stable CT26-FL3 cell line demonstrated great cytotoxicity, as the IC_50_ for quercetin was reduced from 148 µM to 8 µM. The administration of micelles to CRC mouse model, generated by CT26-FL3 cell line injection, determined a decrease in tumor volume and no evident sign of metastasis in other organs. Colorectal tissues were analyzed, and a great increase in apoptosis was noted in comparison to untreated mice as consequence of increase in AMPK and decrease in mTOR phosphorylation and reduced anti-apoptotic protein levels. Change in the tumor immune-microenvironment was also observed with decrease in immune suppressive mechanisms and increase in active immune cells as well as change in the profile of cytokines production. Moreover, administration of QA-M micelles led to generation of immune-memory, demonstrating that the QA-M treatment enhanced the immune response against tumor cells reducing tumor volume, which are all very important results for a typology of CRC that is often resistant to typical chemotherapeutic molecules [127].

Another strategy was to generate nanoparticles of quercetin cross-linked to chitosan (CS) that efficiently released quercetin at the pH value of the tumor microenvironment fluid. The use of induced CRC rats demonstrated that administration by enema reduced tumor angiogenesis and mitosis rate and, moreover, increased apoptosis [128].

The challenge of the applied research to fight cancer is also linked to cell chemoresistance by using HCT-8/TAX cell line (taxol and doxorubicin double resistant). Another drug delivery strategy was considered, in which doxorubicin and quercetin were encapsulated in hollow mesoporous silica nanoparticles coated with polydopamine that dissolve at acidic pH value, to which methoxy-polyethylene glycol amine (mPEG-NH_2_) was bound to ameliorate the blood circulation stability. The uptake and retention of doxorubicin from HCT-8/TAX cells was strongly enhanced by co-treatment with quercetin both in free or encapsulated form and this reflected on cytotoxicity that followed the same trend, with encapsulated form less efficient compared to free form, justified by the authors as consequence of a slow drug release. The major efficiency of the combination of the two molecules is probably linked to induced decrease in P-glycoprotein protein level by quercetin that increases doxorubicin concentration inside the cells and, hence, its cytotoxic effect [129].

A newer technology is based on coaxial electrospinning that leads to protection of biomolecules in the core of the fiber that are released when the external layer is solubilized. In the same manner, quercetin was encapsulated in chitosan nanoparticles and by coaxial electrospinning, it was coated with sodium alginate that is swelled in the gastrointestinal tract while chitosan is degraded in colonic tract. The new delivery system that showed good stability was tested on Caco-2 cell line and growth inhibition was observed, linked to induction of apoptosis and arrest in G0/G1 phase of cell cycle [130].

**Figure 2:**
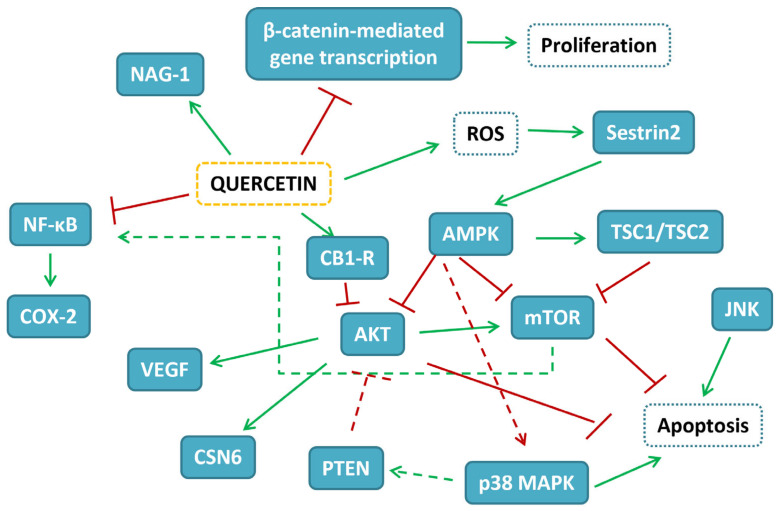
Map of the interactions of quercetin in CRC experimental models. In blue-filled boxes, the protein influenced by quercetin treatment are reported, while blue-dashed boxes indicate cellular events. Red T-shaped lines correspond to inhibition, while green arrows correspond to activation.

The evidence gathered on the potentiality of different delivery systems of quercetin in CRC experimental models is reported in Table 6. The map of the pathways influenced by quercetin in CRC experimental models is depicted in Figure 2.

## 4. Quercetin and Hepatocellular Cancer (HCC)

### 4.1. Quercetin Free Form in HCC Experimental Models

Quercetin was extensively studied as chemotherapeutic molecule against hepatocellular caner (HCC) using principally HepG2 cell line given its morphological characteristics and cellular differentiation resembling normal liver cells. In accordance with this consideration, the utilization of different cell lines has produced heterogeneous results as demonstrated by the study of Hisaka et al., who, using KIM-1, KYN-1, -2, -3, HAK-1A, -1B, -2, -3, -4, -5, and -6, reported different IC_50_ for quercetin in growth inhibition, ranging from 50 to 100 µM, and in apoptosis rate induction, using 100 µM quercetin, ranging from 16.6 ± 1.3% of HAK-4 to 98% of KIM-1. Moreover, cells were blocked in different phases of the cell cycle and the co-treatment with 5-fluorouracil had different effects, ranging from the absence of effect to synergistic one [131]. In agreement with beneficial properties exerted by other flavonols, Zhang et al., using HepG2 cell line treated with quercetin (40 µM for 24 h), reported apoptosis induction and increase in intracellular ROS dependent on upregulation of p53-inducible gene 3 (PIG3) expression, as demonstrated by its silencing that could be responsible for direct oxidation of flavonol [132]. On the contrary, Maurya and Vinayak demonstrated that quercetin treatment that reduced 50% of HepG2 cell viability at 80 µM after 24 h led to progressive decrease in ROS concentration with increase in quercetin concentration [133]. These data are in accordance with those of Jeon et al. [134], who also highlighted a lesser sensibility of Huh-7 compared to that of the HepG2 cell line. The authors noted that quercetin treatment led to decreased PI3K p85α subunit phosphorylation, probably by competing with the binding of ATP to the catalytic site [135], and decrease in total protein kinase C (PKC) activity, as well as PKCα protein level. This reflected on decrease in COX-2 and increase in p53 protein levels, results that were linked, according to the authors, to the inhibition of PKCα that could enhance p53 activity and apoptosis and at the same time decrease COX-2 expression [133]. It must be noted that p53 protein level can also be negatively controlled by PI3K/AKT pathway through mouse double minute 2 homolog (MDM2) E3 ubiquitin ligase. Moreover, mTORC2 and pyruvate dehydrogenase kinase 1 (PDK1) activities that are dependent on PIP3 produced by PI3K are necessary for PKCα activation, even if other more complex pathways cannot be excluded [136,137,138].

It is evident that cellular response can depend on different things such as cell line, flavonol concentration, time of cell incubation and, more generally, experimental conditions. For example, Granado-Serrano et al., using HepG2 cell line treated with quercetin at 50 µM, demonstrated that the experimental data obtained could be dependent on the time of incubation with flavonol, and, in most cases, the dynamics of protein expression and activity changed in a time-dependent manner. Indeed, with their work, they have delineated the importance of the time kinetics for two transcription factors, NF-κB and AP-1, and different signaling pathways in the quercetin-mediated growth inhibition and apoptosis. The use of an inhibitor of JNK, whose phosphorylation was increased by quercetin treatment, has demonstrated that JNK activation was associated with decreased ERK phosphorylation level, indicating cross-talk between them and opposite cellular functions. Moreover, AKT phosphorylation was decreased by quercetin treatment and seems to be related to JNK activity but only after 4 h of treatment, while phosphorylated p38MAPK increased in the intervals of time considered, independently of JNK. In a similar manner, nuclear translocation of NF-κB was decreased by quercetin treatment in the interval of time considered and was dependent on JNK, while activity and nuclear translocation of AP-1 that were increased by quercetin were dependent on JNK-mediated c-Jun activation only in the short interval of time considered. In a similar manner, pro- and anti-apoptotic protein profiles followed the same trend of AP-1 nuclear localization and were differently expressed only after 18 h, an interval of time in which quercetin cell treatment induced 43% of cell death [139].

Apoptosis induction is at the basis of cellular growth inhibition mediated by quercetin, but, using HCC cell line LM3, which is sensitive to quercetin growth inhibition (IC_50_ about 90 µM after 48 h), it was demonstrated that apoptosis is probably preceded by autophagy. Indeed, light chain 3 (LC3) II formation and beclin-1 expression were considered at 24 h while typical apoptotic protein profile was observed after 48 h. Moreover, apart from apoptosis induction, a decrease in invasiveness, EMT and migration was also observed, and, analyzing the mechanism, it was discovered that quercetin reduced Janus kinase 2 (JAK2) and STAT3 phosphorylation levels, even when cells were treated with IL-6, and this reflected on all the cellular responses observed. The in vitro results were confirmed using HCC xenograft mouse model in which the administration of quercetin (100 mg/kg) led to the same response observed in LM3 cell line, as well as decrease in tumor liver volume [140].

The results reported above were partly confirmed by another study based on SMMC-7721 and HepG2 cell lines that showed different IC_50_ in growth inhibition compared to quercetin (21 and 34 μM, respectively) after 48 h. Analysis of SMMC-7221 cells, treated with 40 µM of quercetin for 24 h, clearly demonstrated that it induced autophagy by inhibition of AKT/mTOR pathway and increase in MAPKs activities. Moreover, in the same experimental conditions, apoptosis was also induced, and this was partly inhibited using autophagy inhibitors, demonstrating that autophagy preceded and was necessary to induce quercetin-mediated apoptosis. All the in vitro results were confirmed in vivo using HCC xenograft mouse model after administration of 60 mg/kg of quercetin, demonstrating again the reliability of the in vitro study, as well as the efficacy of quercetin also in vivo [141]. In support of the anti-metastatic effects of quercetin, a study using Huh-7 cell line demonstrated that administration of quercetin at the maximum concentration of 7 µM before HGF (hepatocyte growth factor) or TNF-α treatment reduced cell migration by a mechanism that did not involve their respective receptors or p38MAPK activation, but PI3K/AKT pathway, as demonstrated at a higher quercetin concentration (30 µM) to which its activity was reduced with concomitant increase in E-cadherin protein level [142].

The great interest towards quercetin beneficial effects have permitted to discover other proteins whose expression can be regulated by this flavonol. An example is hexokinase-2 (HK2), which is overexpressed in HCC. Using Bel-7402 and SMMC-7721 cell lines, it was demonstrated that quercetin inhibited cell viability at concentrations lesser than 50 µM, and at this concentration, a reduction in glucose uptake and lactate production was also observed. This effect seems to be linked to downregulation of HK2 protein level, whose expression is controlled positively by PI3K/AKT/mTOR pathway [143,144]. It has to be noted that Li et al., using HCC cell lines, had previously observed that the overexpression of HK2 could also be controlled over PI3K/AKT/mTOR pathway by STAT3, and it is not excluded that in response to different stimuli, both can control this important enzyme [145]. The results obtained in vitro by Wu et al. were also confirmed in vivo using HCC xenograft mouse model by the administration of 50 mg/kg of quercetin, emphasizing the importance of the results [143].

Another group of proteins whose expression can be influenced negatively by quercetin is insulin-like growth factor-2 binding proteins (IGF2BPs), highly expressed in HCC. Using Huh-7 cell line, it was highlighted that quercetin treatment at the concentration of 25 µg/mL (82.7 µM) increased the expression of miR-1275, normally downregulated in HCC that targeted IGF2BPs mRNAs, leading to their degradation [146]. Moreover, in HepG2 cell line, quercetin treatment reduced cell viability with IC_50_ of 12.9 µM, as well as downregulated mRNA and protein level of Sp1, a transcription factor that positively regulates cell cycle progression and inhibits apoptosis [147].

Quercetin can also influence proteasomal activity as demonstrated by a study in which HepG2 cell line was treated at 50 µM of the flavonol and corresponded approximately to the IC_50_ at 48 h, which also induced apoptosis. At this concentration, quercetin also influenced chymotrypsin-like proteasomal activity, and this effect seemed to be linked to ERK1/ERK2 decreased activity that, in turn, led to decreased expression of proteasome β subunits and, in particular, of β5 that is responsible for chymotrypsin-like activity. On the contrary, even if increase in p38MAPK and JNK phosphorylation was observed, the inhibition of these kinases has not affected total proteasomal activity [148].

Apart from the different proteins and pathways that can be influenced by quercetin using HepG2 cell line treated for 48 h at the concentration of 40 µM of the flavonol, great morphological cellular changes were observed as aggregation of F-actin, cytoskeleton disruption and membrane perturbation using atomic force microscopy (AFM), which probably reflects on membrane protein aggregation. Moreover, cells appeared shrunk with numerous apoptotic bodies, and stiffness increased with quercetin increasing concentration, effects probably linked to the deleterious perturbation of cytoskeleton and membrane holes formation that led to loss of osmotic cellular regulation [149].

Most of the results reported above are derived from in vitro studies, even if some of them were also confirmed in vivo. In support of beneficial effects of quercetin in vivo, a study conducted on HCC-induced mice has demonstrated that oral administration of curative (100 mg/mL after HCC induction) or protective quercetin (25 mg/mL during HCC induction) was able to normalize hepatic enzymes dosage and to reduce liver oxidative stress. To better understand the quercetin mechanism of action, CK2-α, Notch and Hedgehog pathways that are normally activated in HCC were considered, and it was confirmed that quercetin administration decreases their expression both at curative or protective dosage, influencing expression levels of proteins involved in cell cycle progression [150].

Data on the anti-cancer effects of quercetin in HCC experimental models are reported in Table 7.

### 4.2. Effects of Combination of First-Line Treatments with Quercetin in HCC Experimental Models

Quercetin was also extensively used as co-adjuvant of chemotherapeutic molecules as for sorafenib, and when HepG2 and Hep3B cell lines were used, it was demonstrated that the combination or pre-treatment with quercetin lowered the concentration of sorafenib necessary to induce 50% of growth inhibition [151]. Moreover, treatment of HepG2, MDBK and Huh-7 cell lines with combination of quercetin and celecoxib proved to be very efficient in terms of growth inhibition and apoptosis induction [152], as well as for combination with 5-fluorouracil, as tested in SMCC-7721 and HepG2 cell lines or HCC xenograft mouse model. In vitro, quercetin treatment at the concentration of 100 µM, when used in combination with 5-fluorouracil 10 µM, led to a decrease in cell growth by apoptosis induction that was much more evident compared to the two molecules used separately. The cooperation between these two molecules was also partly demonstrated in vivo by co-administration, leading to reduction in tumor volume [153].

In the same manner, in HepG2 cell line treated with quercetin at 50 µM, cisplatin (10 µM) anti-tumoral activity could be enhanced by inducing growth inhibition and apoptosis [154].

Similarly, the same concentration of quercetin in combination with roscovitine (10 µM), a CDKs inhibitor tested in HepG2 and Hep3B cell lines, induced growth inhibition in a time-dependent manner and depended on decreased AKT phosphorylation that reflected on pro-apoptotic protein levels increase [155]. Quercetin was also used in combination with gemcitabine (GEM) in GEM-resistant HepG2 cell line and it was demonstrated that the co-treatment with 200 nM GEM and 50 µM quercetin decreased cell viability, and higher apoptosis rate was observed compared to the two single molecules used separately [156].

All the in vitro studies reported are based on the use of the traditional 2D cell culture model that shows well-known limits, thus encouraging a more extensive use of 3D models [157], since the microenvironment parameters are deeply different compared to 2D ones. For these reasons, Hassan et al. used a 3D model of HepG2 cell line treated with doxorubicin 10 μM and quercetin 50 μM, alone or in combination, which showed a strong synergistic effect when the two molecules were co-administered to induce apoptosis in both 2D and 3D cultures; yet in this last condition, the rate of apoptosis observed was higher due to decrease in HIF-1α and increase in both p53 and caspase-3 protein levels linked to a minor activity of MDR1, suggesting anti-oxidative effects of quercetin [158].

To ameliorate the efficacy of chemotherapeutic treatments, quercetin was also tested in combination with ZD55 adenovirus that contains tumor necrosis factor-related apoptosis-inducing ligand (TRAIL) gene insertion in SMMC-7721, HepG2 and Huh-7 cell lines. It was demonstrated that cell viability was reduced to a greater extent compared to the single treatments due to an antagonizing effect on TRAIL-induced NF-κB activation that reduced its protein level, a phenomenon that also affected the expression profile of proteins involved in apoptosis. Moreover, using HCC xenograft mouse model, the combination of ZD55 and quercetin was more effective in reducing tumor volume and in increasing animal survival [159]. Moreover, quercetin (1–10 µM) treatment in HepG2 and Huh-7 cell lines also incremented the anti-proliferative effect exerted by interferon-α, as well as anti-metastatic properties, inhibiting Src homology domain 2 containing tyrosine phosphatase-2 (SHP2) phosphatase activity responsible of STATs and JAK dephosphorylation and inactivation [160].

The evidence collected regarding the synergistic effects of quercetin in HCC experimental models is reported in Table 8.

### 4.3. Delivery Systems for Quercetin in HCC Experimental Models

Different delivery systems were considered to ameliorate the bioavailability, short half-life, and dose–response of quercetin. For example, by using the HepG2.2.15 cell line (HepG2 transfected with hepatitis B virus), it was demonstrated that encapsulated quercetin and superparamagnetic iron oxide nanoparticles into micelles decreased the concentration needed to arrest cell cycle [161], while encapsulated quercetin in nanoparticles formed by PLGA decorated with chitosan and polyethylene glycol (PEG) were used in HepG2 cell line demonstrating a stronger reduction in cell viability by induction of apoptosis compared to free quercetin treatment, with IC_50_ reduced from >100 to 34.69 µM [162].

Another type of encapsulated quercetin tested was in solid lipid nanoparticles constituted by cholesterol that, when assayed on HepG2, led to a more efficient reduction in cell growth compared to free-form quercetin, probably due to a better cellular uptake [163]. Moreover, modified lipid nanoparticles coated with RGD (arginine-glycine-aspartic acid) were used to deliver sorafenib and quercetin (RGD-SRF-QT NPs) in HepG2 cell line and in HCC xenograft mouse model, demonstrating that administration of the nanoparticles is able to inhibit cell growth and reduce tumor volume [164].

Using PLGA encapsulated gold quercetin nanoparticles in MHCC97H, Hep3B, HCCLM3 and Bel7402 cell lines, an increase in growth inhibition was demonstrated proportional to quercetin nanoparticle concentration (maximum tested 60 µM). The effect exerted by quercetin nanoparticles was dependent on reduction in both PI3K/AKT and MEK/ERK pathway activities that were involved in apoptosis induction, reduced hTERT level, inhibition of COX-2 expression, that were also linked to reduced NF-κB nuclear translocation, and downregulation of AP-2β signaling. Moreover, using MHCC97H cell line, a decrease in cellular invasiveness and migration was observed. The treatment of HCC xenograft mouse model with quercetin nanoparticles reduced tumor volume by the same mechanisms highlighted in vitro [165].

**Figure 3:**
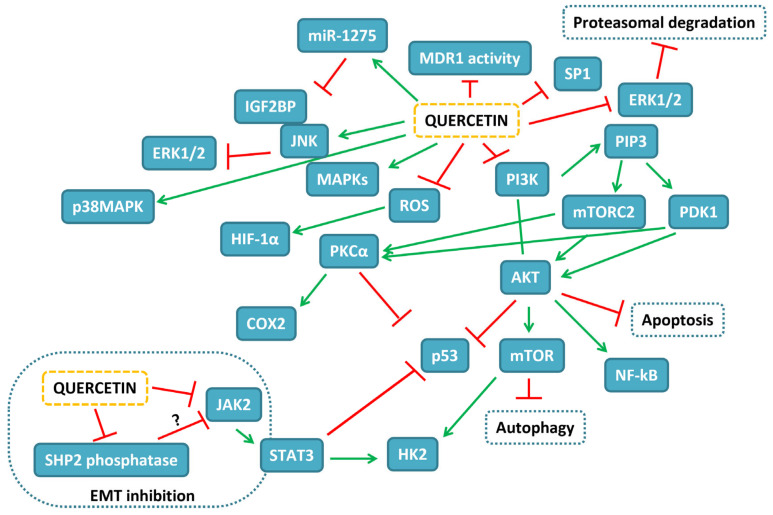
Map of the interactions of quercetin in HCC experimental models. In blue-filled boxes, the proteins influenced by quercetin treatment are reported, while blue-dashed boxes indicate cellular events. Red T-shaped lines correspond to inhibition, while green arrows correspond to activation.

The potentiality of different delivery systems of quercetin in HCC experimental models is reported in Table 9. The map of the pathways influenced by quercetin in HCC experimental models is depicted in Figure 3.

## 5. Conclusions

Different phases of the carcinogenic process can be targeted by phytochemicals, and an increasing number of studies suggests that these compounds may be useful for both prevention and as co-adjuvants in anti-cancer therapy. This is because compared to synthetic drugs, their lower specificity is their forte since several targets can be simultaneously influenced by phytochemicals. Quercetin stands out among the other flavonoids given a plethora of its documented pharmacological activities, including anti-cancer one. In this review, we gathered the evidence on the ability of this flavonol to act at different stages of tumorigenesis, as reviewed, resulting in a great candidate drug. Indeed, quercetin has shown to target receptors, enzymes, and transcriptional factors crucial in the initiation, promotion, and progression of cancer. Moreover, its role of sustaining the effect of well-established chemotherapeutic agents is of great interest, thus potentially allowing a reduction in their dosages. Noteworthy is the fact that the exploitation of novel delivery systems can overcome quercetin’s poor water solubility, chemical instability, and poor bioavailability, hence accelerating its path from bench to bedside. Therefore, further studies are essential, especially in more complex models, to thoroughly define the potentiality of this compound to eventually occupy its rightful place in the fight against the great burden of cancer.

## Figures and Tables

**Table 1:** Targets affected by quercetin free form in BC experimental models.

Cell [or Animal] Model	Concentration	Effect	Reference
MDA-MB-468	23–55 µM	Arrest of cells in G2/M phase and growth inhibition	[36]
MCF-7	17.2 µM	Reduction in cell growth	[37]
MCF-7	4.9 µM	Counteractive effects on pro-proliferative effects of E2 and TNF- α	[38]
MCF-7	1–20 µM	Induction of apoptosis and arrest of cells in G2/M phase (p21 dependent)	[39]
MCF-7	48 µM	Increased ROS production	[40]
MCF-7	100 µM	Increased ROS production; induction of apoptosis; AMPK activation and decrease of COX-2 protein levels	[41]
MCF-7	100 µM	Reduction in proliferation	[42]
MCF7, T47D (ER^+^) MDA-MB-231, HCC-38 (ER^−^)	0.1–60 µM	(ER^+^): pro-proliferative effects at lower concentrations; anti-proliferative effects at higher concentrations (ER^−^): anti-proliferative effects	[43]
MCF-7, MDA-MB-231	5–20-100 µM	Lower concentrations: pro-proliferative effects Higher concentrations: anti-proliferative effects	[44]
MCF-7, SK-Br-3	100 µM	Induction of c-fos; activation of MEKs and ERK1/2; EGFR and MAPK activation	[45]
MCF-7	0.1–1000 nM	Increase in PTEN protein level; decrease in phosphorylated AKT; increase in p27; arrest of cell cycle	[46]
HCC1937 (PTEN^−/−^), T47D (PTEN^+/+^)	25 µM	Reduction in phosphorylated AKT level	[47]
MCF-7, MDA-MB-231	30 µM [50 mg/kg]	Decrease in cellular invasion and migration capacities; decrease in AKT/mTOR activity; autophagy induction; decrease in GLUT1, PKM2, LDHA; reduction in tumor volume	[48]
BCSC (CD44^+^ from MCF-7) [xenograft]	50 µM	Reduction in cell viability and metastatic properties; reduction in tumor volume	[50]
HCC1806, HCC70, HCC1937, BT-549, BT-20, Hs578T, MDA-MB231, MDA-MB 157 and MDA-MB-468 BT-549	200 µM	Migration inhibition and decreased invasion abilities; decreased AKT phosphorylation; decrease in GSK3α/β and WNK-1; reduced phosphorylation of β-catenin, ERK1/2, JNK1/2/3, p38α; increase in CHK2 phosphorylation	[51]
MDA-MB-231, MDA-MB-435	15 µM	Increase in AMPK phosphorylation; decrease in AKT activity; growth inhibition and arrest of cell cycle in G2/M phase	[52]
MDAMB-231, MDA-MB-157	230, 415 µM	Reduced lipid synthesis; inhibition of FAS; reduction of cell viability and induction of apoptosis; decrease in FASN, β-catenin, Bcl-2	[55]
MCF-7		Increased level of phosphorylated AMPK; decreased level of phosphorylated AKT	[57]
MDA-MB-231	20 µM	Decreased cell viability; increased apoptosis; cell cycle arrest; JNK and FOXO3a increase	[58]
MCF-7, MDA-MB-231 [xenograft]		Induction of apoptosis; negative regulation of EGFR; increase in miR-146a expression; reduction in tumor volume	[59]
MCF-7, MDA-MB-231	100 µM	Reduction in HSP70 and HSP27, HSP90 protein levels	[60,61]
BCSC ALDH^+^, AS-B145, AS-B244	0–200 µM	Reduction in HSP27; decrease in mammosphere dimension, cell migration and EMT; decrease in nuclear translocation of NF-κB and proteasomal degradation of IκBα HSP27-mediated	[63]
MDA-MB-231	150 µM	Growth inhibition; decrease in intracellular calcium concentration; decrease in urokinase activity	[64]
TNBC MDA-MB-231	25 µM	Reduction in PFKP and LDHA protein levels; decrease in cellular invasiveness and migration	[65]

AKT: AKT serine/threonine kinase; AMPK: AMP-activated protein kinase; BCSC: breast cancer stem cells; CHK: checkpoint kinase; COX-2: cyclooxygenase 2; EGFR: epithelial growth factor receptor; EMT: epithelial–mesenchymal transition; ER: estrogen receptor; ERK extracellular signal-regulated kinase; FAS: fatty acid synthase; FOXO3a: forkhead box O3A; GLUT1: glucose transporter 1; GSK: glycogen synthase kinase; HSP; heat shock protein; JNK: c-JUN terminal kinase; LDHA: lactate dehydrogenase A; MAPK: mitogen-activated protein kinases; MEK: MAPK kinase; mTOR: mammalian target of rapamycin; PFKP: phosphofructokinase platelet-type; PKM2: pyruvate kinase M2; PTEN: phosphatase and tensin homolog; ROS: reactive oxygen species; TNCB: triple negative breast cancer; TNF-α; tumor necrosis factor alpha; WNK-1: WNK lysine deficient protein kinase 1.

**Table 2:** Effects of co-administration of quercetin with first-line chemotherapeutic agents in BC experimental models.

Cell [or Animal] Model	First-Line Agent	Effect of Combination	Reference
MCF7-DR	Docetaxel	Synergistic increase in cytotoxicity; decrease in Lef1	[67]
MDA-MB-231	Docetaxel	Synergistic induction of apoptosis; increase in p53 and BAX; decrease in pAKT, pERK1/2, pSTAT3	[68]
EMT6 [xenograft]	Cisplatin	Greater cytotoxic effects; reduction in tumor volume	[69]
MDA-MB-231	5-fluorouracil	Greater decrease in cell viability and migration; decrease in MMP-2 and -9 expression	[70]
MCF-7	5-fluorouracil	Synergistic increase in apoptosis	[71]
MCF-7, BT-20	rhTRAIL	Enhancement of apoptosis; induction of proteasomal degradation; reduction in c-FLIP and increase in DR5	[72]
MCF-7/ADR	Paclitaxel	Downregulation of P-glycoprotein	[73]
MCF-7/ADR	Doxorubicin, paclitaxel, vincristine	Enhancement of cytotoxic effects; decrease in P-glycoprotein protein level and YB-1 nuclear translocation	[74]

BAX: BCL2-associated X protein; c-FLIP: CASP8 and FADD-like apoptosis regulator cellular FADD-like IL-1β-converting enzyme-inhibitory protein; DR5: Death Receptor 5; Lef1: lymphoid enhancer-binding factor 1; MMP: matrix metallopeptidase; rhTRAIL: human recombinant tumor necrosis factor-related apoptosis-inducing ligand; YB-1: Y-box binding protein 1.

**Table 3:** Targets affected by quercetin in different delivery systems in BC experimental models.

Cell [or Animal] Model	Delivery System	Effect	Reference
MCF-7/ADR	Encapsulated quercetin and paclitaxel in MSNs-ChS@PQ	Augmented cytotoxicity and apoptosis	[75]
MDA-MB-231/MDR1	Encapsulated quercetin and doxorubicin in mixed micelles of HA-based conjugate and d-α-tocopheryl poly-(ethylene glycol) 1000 succinate	More efficient induction of apoptosis	[76]
4T1 [xenograft]	Encapsulated quercetin in nanoparticles of RSF coated with LyP-1-QU-NPs	Greater inhibition of cell viability; stronger apoptosis induction; reduced tumor volume	[77]
MCF-7	Nanoparticles of apoferritin loaded with quercetin and curcumin	Increase in ROS production and apoptosis induction	[79]
MCF-7	Solid lipid nanoparticles loaded with quercetin and curcumin	Increase in ROS production and apoptosis induction	[80]
4T1	Nanoparticles formed by PLGA, linked to PEI, and bound to HA of quercetin and docetaxel	Decrease in phosphorylated AKT and MMP-9 protein level; decrease in NF-κB activity	[81]
MCF-7, MDA-MB-231	Quercetin-conjugated gold nanoparticles	Decreased EMT, migration and invasion abilities; strong inhibition of PI3K/AKT pathway; reduction in EGFR activity	[85]

HA: hyaluronic acid; MSNs-ChS@PQ: mesoporous silica nanoparticles coated with chondroitin sulfate; PEI: polyethyleneimine; PLGA: polylactic-co-glycolic acid; RSF: regenerated silk fibroin.

**Table 4:** Targets affected by quercetin free form in CRC experimental models.

Cell (or Animal) Model	Concentration	Effect	Reference
SW480/ mouse CRC clone 26	60/160 µM	Reduction in cell growth; cell cycle blockage	[87]
Caco-2	5–50 µM	Reduction in cell growth; downregulation of cell cycle-related factor mRNAs	[88]
HT-29	15 µM	Cell growth inhibition	[86]
HT-29, HCT-116	0–70 µM	Pro-proliferative effects	[42]
HT-29, COLO205, COLO205-X	200 µM	Reduction in cell viability	[89]
HT-29 [xenograft]	100 µM [50–100 mg/kg]	Growth inhibition; chromatin condensation; cell cycle arrest in G1 phase; AMPK activation; induction of apoptosis	[91]
HT-29	1–100 µM	COX-2 protein level decrease; induction of apoptosis; increase in IκBα expression	[41,92]
Caco-2, SW620	35–20 µM	Growth inhibition; induction of caspase-dependent apoptosis; reduction in p65 and IκBα phosphorylated protein levels; increase in IκBα expression	[93]
HCT116, HT-29	25–50 µM	ROS production; apoptosis induction; increase in sestrin2	[96,98]
DLD-1	1 µM	Induction of apoptosis by activation of ERβ1; increased p38MAPK phosphorylated and PTEN expression; PI3K/AKT/mTOR pathway activation	[101]
HCT116 [xenograft]	100 µM [50 mg/kg]	Apoptosis induction; inhibition of AMPK activity; reduction in tumor volume and AMPK phosphorylation	[102]
HT-29	81.65 µM	Induced strong cellular morphological changes and apoptosis; modulation of CSN6 activity	[103]
HT-29, SW480	100 µM	Induction of apoptosis; decrease in ErbB2 (HER2) and 3 (HER3); decrease in PI3K activation	[104]
Caco-2, DLD-1	50 µM	Up-regulation of CB1-R; inhibition of growth; inhibition of PI3K/AKT activity; JNK and c-Jun activation	[105,106]
[AOM/DSS-induced CRC]	[30 mg/kg]	Decrease in tumor size and volume	[107,108]
HCT-116	40 µM	Increase in NAG-1 expression; increase in SP1 and EGR-1 transcription factors	[109]
DLD-1	20 µM	TNF-alpha-stimulated COX-2 expression	[110]
Caco-2	5 µM	Inhibition of TLR-4 and NF-κB activity	[111]
SW480	100 µM	Inhibition of TGF-β1-induced EMT; increase in E-cadherin; decrease in vimentin and Twist1	[112]
HCT-15, CO-115	20 µM	Apoptosis induction; inhibition of RAS and PI3K activity	[114]
SW480, HCT116, DLD-1^KRASG13D^	100 µM	Induction of extrinsic and intrinsic apoptosis; inhibition of AKT and activation of JNK	[115]

CSN6: COP9 signalosome subunit 6; EGR-1: early growth response 1; NAG-1: non-steroidal anti-inflammatory drug-activated gene-1; SP1: specificity protein 1; TLR-4: toll-like receptor 4.

**Table 5:** Effects of co-administration of quercetin with first-line chemotherapeutic agents in CRC experimental models.

Cell [or Animal] Model	First-Line Agent	Effect of Combination	Reference
HCT-15	5-fluorouracil	Enhancement of caspase-independent apoptosis	[120]
HT-29	5-fluorouracil	Increase in apoptosis induction	[121,126]
HT-29	5-fluorouracil	Increase in p53 expression; decrease in AKT and mTOR pathways	[122]
DLD-1 [Xenograft]	Ionizing radiation	Reduction in tumor volume	[123]
HT-29	Cisplatin	Reduction in cell viability; induction of apoptosis and cell cycle blockage	[124]
CSC	Doxorubicin	Enhancement of cytotoxic activity and cell arrest in G2/M phase	[124,125]

**Table 6:** Targets affected by quercetin in different delivery systems in CRC experimental models.

Cell [or Animal] Model	Delivery System	Effect	Reference
CT26-FL3 [xenograft]	Encapsulation of quercetin and alantolactone in micelles of DSPEPEG2000 and TPGS	Greater cytotoxicity (IC_50_ drop from 148 to 8 µM); reduction in tumor volume; decrease in mTOR phosphorylation	[127]
[CRC rats]	Nanoparticles of quercetin cross-linked to chitosan	Reduction in tumor angiogenesis and mitosis rate; increase in apoptosis	[128]
HCT-8/TAX	Encapsulated doxorubicin and quercetin in hollow mesoporous silica nanoparticles, coated with polydopamine bound to mPEG-NH_2_	Better uptake; decrease in P-glycoprotein protein level	[129]
Caco-2	Encapsulated quercetin in chitosan nanoparticles coated with sodium alginate by coaxial electrospinning	Increase in growth inhibition; induction of apoptosis; arrest in G0/G1 phase	[130]

DSPEPEG2000: 1,2-distearoyl-sn-glycero-3-phosphoethanolamine-N-methoxy-polyethylene glycol 2000; mPEG-NH_2_: methoxy-polyethylene glycol amine; TPGS: D-α-tocopherol polyethylene glycol succinate.

**Table 7:** Targets affected by quercetin free form in HCC experimental models.

Cell (or Animal) Model	Concentration	Effect	Reference
KIM-1, KYN-1, -2, -3, HAK-1A, -1B, -2, -3, -4, -5, and -6	50–100 µM	Growth inhibition; apoptosis induction; cell cycle blockage	[131]
HepG2	40 µM	Apoptosis induction; increase in intracellular ROS dependent on upregulation of PIG3 expression	[132]
Huh-7, HepG2	80 µM	Decrease in ROS; decrease in PI3K p85α subunit phosphorylation, total PKC activity, PKCα and COX-2 protein level; increase in p53	[133,134]
HepG2	50 µM	JNK activation; decreased ERK and AKT phosphorylation level; decreased nuclear translocation of NF-κB; increased nuclear translocation of AP-1	[139]
LM3 (Xenograft)	90 µM (100 mg/kg)	Growth inhibition; autophagy and apoptosis induction; decrease in invasiveness, EMT and migration; reduced JAK2 and STAT3 phosphorylation levels	[140]
SMMC-7721, HepG2 (Xenograft)	40 µM (100 mg/kg)	Autophagy induction by inhibition of AKT/mTOR pathway and increase in MAPKs activities	[141]
Huh-7	7–30 µM (+HGF or +TNF-α)	Reduced cell migration, PI3K/AKT pathway; E-cadherin increase	[142]
Bel-7402, SMMC-7721 (Xenograft)	<50 µM (50 mg/kg)	Inhibition of cell viability, downregulation of HK2	[143]
Huh-7	82.7 µM	Increased expression of miR-1275; degradation of IGF2BPs mRNA	[146]
HepG2	12.9 µM	Downregulation of Sp1	[147]
HepG2	50 µM	Induction of apoptosis; influence of chymotrypsin-like proteasomal activity; ERK1/ERK2 decreased activity; decrease in proteasome β expression	[148]
HepG2	40 µM	Increase in aggregation of F-actin, cytoskeleton disruption and membrane perturbation	[149]
(HCC-induced mice)	(100 or 25 mg/mL, before or after HCC induction, respectively)	Normalization of hepatic enzymes; reduction in liver oxidative stress; decrease in CK2-α and Notch and Hedgehog pathways	[150]

CK2-α: casein kinase 2 alpha; HGF: hepatocyte growth factor; HK2: hexokinase 2; IGF2BP: insulin-like growth factor-2 binding proteins; PI3K: phospho-inositol-3 kinase; PIG3: p53-inducible gene 3.

**Table 8:** Effects of co-administration of quercetin with first-line chemotherapeutic agents in HCC experimental models.

Cell [or Animal] Model	First-Line Agent	Effect of Combination	Reference
HepG2, Hep3B	Sorafenib	Combination or pre-treatment with quercetin lowered sorafenib IC_50_	[151]
HepG2, MDBK, Huh-7	Celecoxib	Growth inhibition and apoptosis induction	[152]
SMCC-7721, HepG2 (Xenograft)	5-fluorouracil	Decrease in cell growth by apoptosis induction; reduction in tumor volume	[153]
HepG2	Cisplatin	Induction of growth inhibition and apoptosis	[154]
HepG2, Hep3B	Roscovitine	Induction of growth inhibition; decrease in AKT phosphorylation; pro-apoptotic protein levels increase	[155]
HepG2/GEM	Gemcitabine	Decrease in cell viability and higher apoptosis rate	[156]
HepG2 (3D)	Doxorubicin	Induction of apoptosis	[158]
SMMC-7721, HepG2, Huh-7 (Xenograft)	ZD55 adenovirus	Reduction in cell growth and tumor volume	[159]
HepG2, Huh-7	Interferon-α	Increase in anti-proliferative effects; inhibition of SHP2	[160]

GEM: gemcitabine; SHP2: Src homology domain 2 containing tyrosine phosphatase-2.

**Table 9:** Targets affected by quercetin in different delivery systems in HCC experimental models.

Cell (or Animal) Model	Delivery System	Effect	Reference
HepG2.2.15	Encapsulated quercetin and superparamagnetic iron oxide nanoparticles into micelles	Decrease in the concentration needed to arrest cell cycle	[161]
HepG2	Encapsulated quercetin in nanoparticles formed by PLGA decorated with chitosan and PEG	Stronger reduction in cell viability compared to quercetin free form; induction of apoptosis	[162]
HepG2	Encapsulated quercetin in solid-lipid nanoparticles of cholesterol	More efficient reduction in cell growth compared to free-form quercetin	[163]
HepG2 (Xenograft)	Encapsulated quercetin and sorafenib in modified lipid nanoparticles coated with RGD	Inhibition of cell growth; reduction in tumor volume	[164]
MHCC97H, Hep3B, HCCLM3 and Bel7402	PLGA encapsulated gold quercetin nanoparticles	Increase in growth inhibition; reduction in both PI3K/AKT and MEK/ERK pathway activities; hTERT reduced level; inhibition of COX-2 expression; reduction in NF-κB nuclear translocation; downregulation of AP-2β signaling; reduced tumor volume	[165]

PEG: polyethylene glycol; RGD: arginine–glycine–aspartic acid.

## Data Availability

Not applicable.
